# Transcriptome-wide study revealed m6A regulation of embryonic muscle development in Dingan goose (*Anser cygnoides orientalis)*

**DOI:** 10.1186/s12864-021-07556-8

**Published:** 2021-04-14

**Authors:** Tieshan Xu, Zijie Xu, Lizhi Lu, Tao Zeng, Lihong Gu, Yongzhen Huang, Shunjin Zhang, Peng Yang, Yifan Wen, Dajie Lin, Manping Xing, Lili Huang, Guojun Liu, Zhe Chao, Weiping Sun

**Affiliations:** 1 Institute of Animal Science & Veterinary Medicine, Hainan Academy of Agricultural Sciences, No. 14 Xingdan Road, Haikou, 571100 People’s Republic of China; 2 Tropical Crops Genetic Resources Institute, Chinese Academy of Tropical Agricultural Sciences, Haikou, 571101 People’s Republic of China; 3 College of Animal Science and Technology, Northwest A&F University, Yangling, Shaanxi 712100 People’s Republic of China; 4 Institute of Animal Husbandry and Veterinary Science, Zhejiang Academy of Agricultural Sciences, Hangzhou, People’s Republic of China; 5 Key Laboratory of Tropical Animal Breeding and Disease Research, Haikou, 571100 People’s Republic of China; 6 Institute of Animal Husbandry of Heilongjiang Academy of Agricultural Sciences, Haerbin, Heilongjiang 150086 People’s Republic of China

**Keywords:** *Anser cygnoides orientalis*, Breast muscle tissues, m6A-sequencing, Differentially methylated genes, miRNAs-sequencing

## Abstract

**Background:**

The number of myofiber is determined during the embryonic stage and does not increase during the postnatal period for birds, including goose. Thus, muscle production of adult goose is pre-determined during embryogenesis. Previous studies show *N*^*6*^-methyladenosine (m6A) is an important regulator for skeletal muscle development of birds and miRNAs play as a co-regulator for the skeletal muscle development in birds. Herein, we sequenced m6A and miRNA transcriptomes to investigate the profiles of m6A and their potential mechanism of regulating breast muscle development in Dingan Goose.

**Results:**

We selected embryonic 21th day (E21) and embryonic 30th day (E30) to investigate the roles of transcriptome-wide m6A modification combining with mRNAs and miRNAs in goose breast muscle development. In this study, m6A peaks were mainly enriched in coding sequence (CDS) and start codon and397 genes were identified as differentially methylated genes (DMGs). GO and KEGG analysis showed that DMGs were highly related to cellular and metabolic process and that most DMGs were enriched in muscle-related pathways including Wnt signaling pathway, mTOR signaling and FoxO signaling pathway. Interestingly, a negative correlation between m6A methylation level and mRNA abundance was found through the analysis of m6A-RNA and RNA-seq data. Besides, we found 26 muscle-related genes in 397 DMGs. We also detected 228 differentially expressed miRNAs (DEMs), and further found 329 genes shared by the target genes of DEMs and DMGs (m6A-miRNA-genes), suggesting a tightly relationship between DEMs and DMGs. Among the m6A-miRNA-genes, we found 10 genes are related to breast muscle development. We further picked out an m6A-miRNA-gene, PDK3, from the 10 genes to visualize it and the result showed differentially methylated peaks on the mRNA transcript consistent with our m6A-seq results.

**Conclusion:**

GO and KEGG of DMGs between E21 and E30 showed most DMGs were muscle-related. In total, 228 DEMs were found, and the majority of DMGs were overlapped with the targets of DEGs. The differentially methylated peaks along with an m6A-miRNA-gene, PDK3, showed the similar results with m6A-seq results. Taken together, the results presented here provide a reference for further investigation of embryonic skeletal muscle development mechanism in goose.

**Supplementary Information:**

The online version contains supplementary material available at 10.1186/s12864-021-07556-8.

## Background

RNA plays numerous critical roles in cellular processes ranging from the transfer of genetic information from DNA to protein or to the epigenetic modulation of gene transcription [1, 2]. In a similar manner to proteins and DNA, chemical modifications could also influence the metabolism, function and localization of RNA. More than 150 diverse chemical groups are known to modify RNA at one or more of its four nucleotides (A, G, C and U) [3]. Among which, methylation of adenosine at the N6 position (m6A) is the most prevalent epigenetic modification of RNAs, which is first reported 50 years ago [4, 5] and contributed to the generation, processing, localization and function of RNAs [6–8].

Recent studies have discovered protein function as ‘erasers’, ‘writers’ and ‘readers’ of m6A chemical marks, which work together and dynamically regulate m6A. Fat mass and obesity-associated protein (FTO) as the first m6A demethylase (eraser) was identified was in 2011 [9]. Soon afterwards, another demethylase (eraser), AlkB homolog 5 (ALKBH5), was found 3 years later in 2014 [10]. The methyltransferase (writers) of m6A always deposited in mRNA as a multicomponent m6A methyltransferase complex, which consists of a core complex, the methyltransferase-like 3 (METTL3) / methyltransferase-like 14 (METTL14) heterodimer, and other regulatory component including WTAP, KIAA1429, ZC3H13 and RBM15/15B [11–16]. Differing from the function as‘erasers’ and ‘writers’, m6A-binding proteins (readers), which preferentially recognize m6A modification, can bind to methylated m6A site and perform specific functions. For instance, YTH domain-containing family protein 2 (YTHDF2) accelerates mRNA degradation through locating on p-body [17], while YTHDF1 and YTHDF3 promote translating by recruiting initiation factors in Hela cells [18, 19]. miRNAs are a kind of non-coding RNAs that involved in post-transcriptional genes expression and gene silencing. Besides, a previous study indicated that miR-145 modulates the m6A levels in clinical hepatocellular carcinoma (HCC) tissues by targeting the 3’UTR of YTHDF2 mRNA [20].

Several studies have explored the roles of m6A in disease, development and profiling of plants and animals, and other aspects, which suggest the versatile functions of m6A modification. In diseases, the role of m6A was showed in self-renewal and cell fate [21], and control the anti-tumor immunity [22]. In plant, the m6A methylation patterns were explored [23–25]. For a long time, scientists have focused on exploring m6A’s roles to reveal the law of animal tissue development. In animals, Tao et al. (2017) found the m6A methylation was mainly enriched in stop codons, 3′-untranslated regions, and coding regions in porcine muscle and adipose tissues [26]. Lence et al. (2016) investigated the neuronal functions and sex determination in Drosophila modulated by m6A, and pointed out that the nuclear YT521-B protein may be a key effector for neuronal functions and sex determination [27]. Zhao et al. (2017) found that m6A-dependent maternal mRNA clearance facilitates zebrafish maternal-to-zygotic transition [25]. For birds, Fan et al. (2019) reported the m6A peaks and m6A modified transcripts appearing increasing trend during follicle selection, and further revealed the Wnt pathway may play a vital role in this process [28]. However, the profiling of goose m6A in many tissues, including skeletal muscle, is deficient, which greatly impedes the exploration of m6A mechanism in goose.

In this study, we aimed at investigating the m6A profiles in embryonic breast muscles of Dingan goose and exploring the potential regulation mechanism of m6A cooperating with miRNAs in breast muscle development of goose. Thus, we carried out a transcriptome-wide m6A methylation analysis in embryonic 21th day (E21) and embryonic 30th day (E30) of Dingan goose. The results showed that m6A peak is highly enriched around the CDS and start codon, where contrasting to yeast and mammalian systems [29, 30]. Moreover, out study revealed a negative correlation between m6A modification level and mRNA expression abundance based on potential miRNAs regulation. Finally, 10 potential m6A-miRNA-genes (genes shared by DMGs and DEMs) were picked out in this study and one of which has a methylation difference in the transcript of the PDK3 gene in E21 and E30, which underlying that miRNAs were possibly affected by the m6A levels of key genes and then to regulate the embryonic breast muscle growth in Dingan goose. The results of this paper could improve the understanding of the roles of m6A in goose skeletal muscle development.

## Results

### E21 is the fastest point of breast muscle development during the embryonic stage of Dingan goose

**Fig. 1:**
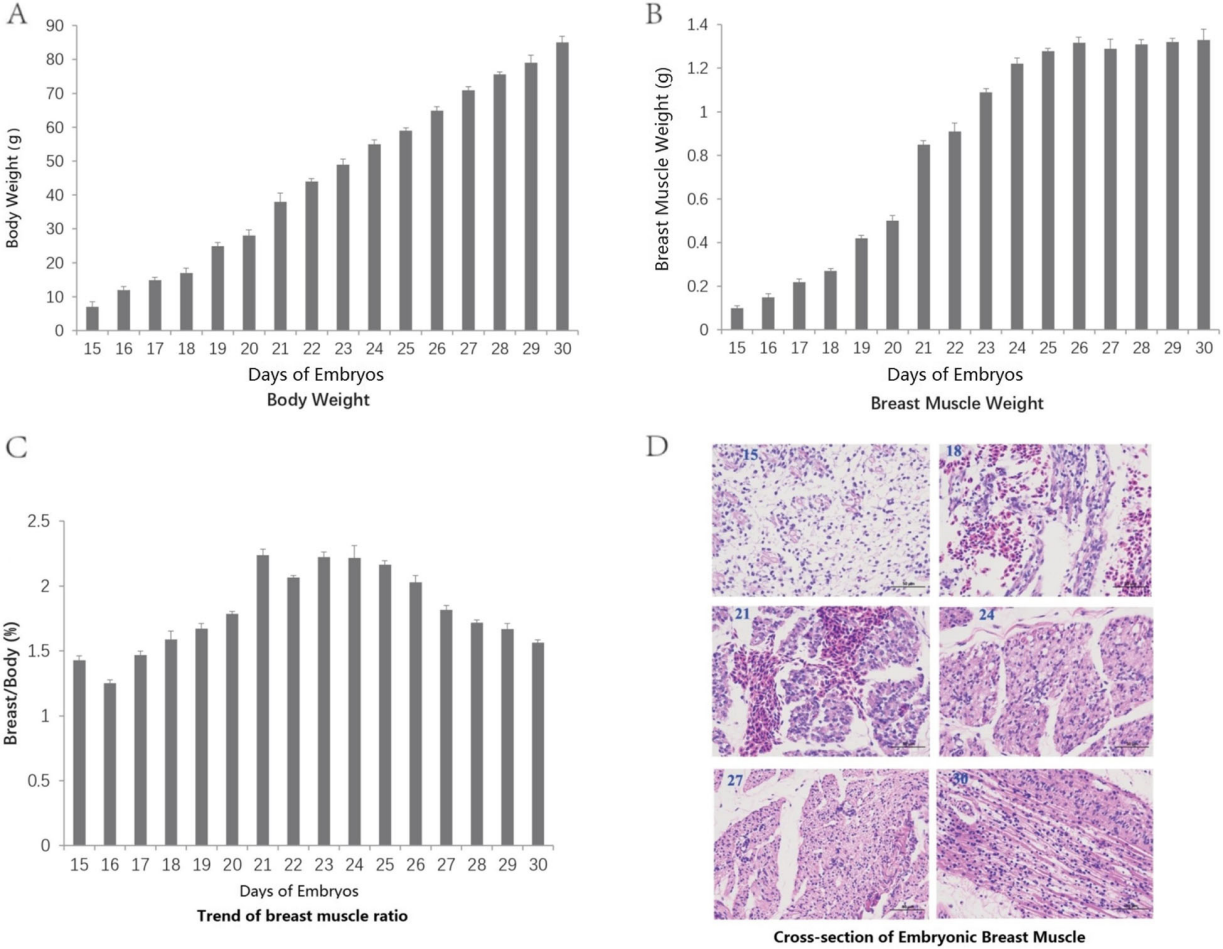
Outline of breast muscle development during the embryonic stages of Dingan goose. **a** Trend of body weights. **b** Trend of breast muscle weights. **c** Trend of breast muscle ratio (breast weight/ body weight*100%). **d** Embryonic breast muscle slices of goose (20 × 20, HE). 15, 18, 21, 24, 27, 30 represented to embryonic 15th day (E15), E18, E21, E24, E27 and E30, respectively

The number of bird skeletal muscle fibers almost fixed during embryonic stage and there are no significant changes in fiber numbers during postnatal stage. Therefore, the research of bird skeletal muscle fiber development in embryonic stage is very important for understanding the development mechanism of bird skeletal muscle and has been focused by many scientists [12]. In this study, we performed anatomical analysis for Dingan goose embryos from E15 to E30 day by day. The results indicated that the embryonic weights increased continuously from E15 to E30 (Fig. 1a), while the breast muscle weights were proportional to body weight changes before E21 and almost ceased after E23 (~ 1.3 g) (Fig. 1b). Thus, the breast muscle rate (breast weight / body weight*100%) increased with age day before E23, and then decreased afterwards (Fig. 1c). Subsequently, we carried out the analysis of embryonic breast muscle using paraffin section method to explore the muscle development process. We found that E15 to E21 mainly involved in muscle fiber proliferation events to form more mono-nucleated fibers. E24 to E30 represented the stage of fusion, to form more multinucleated myotubes, and myotubes bound to the perimysium to form myolin. With the myotubes developing continuously, they already had the same shape at E30 as muscle fibers in adult animal (Fig. 1d). The results above inspired us to explore whether the expression levels of key genes in skeletal muscle regulation changed or not, which would provide a fundamental reference for goose skeletal muscle development. Consistent with this, MSTN gene, an inhibitor of skeletal muscle development [31], was significantly suppressed from E15 to E21 that reached its minimum value at E21 then slowly increased from E21 to E30 in our qRT-PCR assay (Supplementary Fig. S1A). Conversely with MSTN, MyoG and MyoD, which positively regulate muscle growth [32, 33], the expression of MyoG and MyoD showed opposite expression trends and reached the peak values at E27 and E21 respectively (Supplementary Fig. S1B&C). Taken together our results above, we found that E21 is the fastest point of embryonic breast muscle growth for Dingan goose and that E15 and E30 were two different points related to E21 in growth and property of embryonic breast muscle for Dingan goose (Fig. 1a and b). Given m6A modification is the most prevalent epigenetic modification of RNAs and may play as crucial roles in the development of skeletal muscle of goose [26, 28], we selected E21 and E30 to investigate the potential regulation of m6A modification in Dingan goose embryonic skeletal muscle through m6A-seq technology.

### Transcriptome-wide m6A-seq revealed global m6A modification patterns in embryonic breast muscle tissue from *Anser cygnoides orientalis*

In this study, we selected breast muscles of E21 and E30 from Dingan goose for transcriptome-wide m6A-sequencing (m6A-seq) and RNA-sequencing (RNA-seq) assays, with three biological replicates for each group. From m6A-seq, we detected 6.4–7.2 million reads in E21, and about 4.4 million valid reads were mapped to reference genome of *Anser cygnoides orientalis* for each individual (Supplementary Table S1). Similarly, 7.0–8.3 million reads were generated in E30, and about 5.0 million valid reads were mapped for each individual (Supplementary Table S1). For RNA-seq, 9.2–9.3 million reads were generated, and about 4.7 million valid reads were mapped to genome in E21 for each individual (Supplementary Table S1). Respectively, 7.9–9.2 million reads were generated, and about 4.6 million valid reads were mapped to genome in E30 for each individual (Supplementary Table S1). As a result, most of the mapped reads were in the exons. However, due to the alternative splicing situation, there were a few reads mapped to introns (Supplementary Fig. S2).

**Fig. 2:**
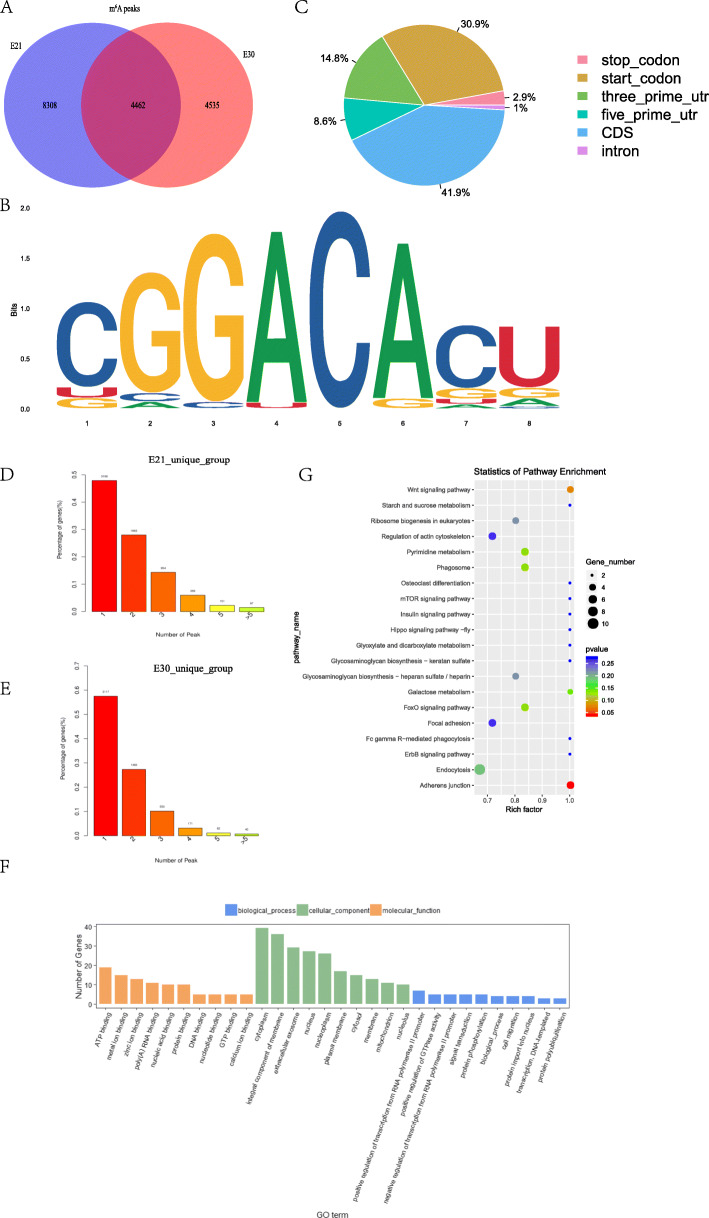
Overview of transcriptome-wide m6A in Dingan goose. **a** Common and unique m6A peaks in E21 and E30. E21 and E30 mean embryonic 15th day (E15) and 30th day, respectively. **b** Motif sequence of m6A contained. **c** Proportion of m6A peaks fallen along transcripts. **d** The m6A peak number covered by a gene in E21. **e** The m6A peak number covered by a gene in E30. **f** GO analysis of differentially methylated genes (DMGs). **g** KEGG analysis of DMGs

We identified 12,770 peaks by R package exomePeak [34] (v 1.8; *P* < 0.05) in E21, representing transcripts of 6650 genes (genes whose transcript carry m6A peaks, abbreviated as m6A genes), and identified 8997 peaks in E30, representing transcripts of 5423 m6A genes (Supplementary Table S1). Among them, there were 4535 E30-unique peaks and 8308 E21-unique peaks (Supplementary Table S2; Fig. 2a).

As a matter of fact, the motif was similarly revealed to be necessary for the process of m6A methylation in mammals and yeast mRNA [29, 30, 35]. Then, we also analyzed the significant peaks (Supplementary Table S3) to identify whether the m6A peaks contained the m6A methyltransferase-combined consensus motifs of RRACH (i.e. R represents purine, A is m6A, C is cytosine and H represents a non-guanine base) [5, 36]. We examined each peak to determine whether it contains a motif in E21 or E30 and the results prove that it does exist (Fig. 2b).

To investigate the preferential location of methyltransferase in transcripts, we subsequently studied the distribution of m6A peaks in the whole transcriptome-wide of E21 and E30 by coordinating the reference genome of *Anser cygnoides orientalis.* We separated a transcript into stop codon, start codon, 3′ untranslated regions (UTR), 5′ UTR, CDS and intron to figure out preferential region that peaks fall. The result showed that peaks were markedly enriched in the CDS and the start codon, following by the 3′ UTR and 5′ UTR for both of the two groups (Fig. 2c), which contrast to the previous m6A study [30]. We also categorized transcript within different numbers of m6A peak for each group. In E21, there were 3380 transcripts of genes only one peak, accounting for nearly 50% (Fig. 2d), and 3238 transcripts with only one peak in E30, accounting for nearly 60% (Fig. 2e). The topological patterns distributing with genes were highly similar in both tissues.

To further analyze general potential function of m6A genes in goose embryonic breast muscle tissues. We scanned all of 418 differentially methylated peaks and found 397 differentially methylated genes (DMGs) (Supplementary Table S4). GO analysis (Supplementary Table S5; Fig. 2f) showed those DMGs were enriched in terms of positive regulation of GTPase activity, protein phosphorylation, ATP binding. It followed that the enrichment of each GO term was different within three ontologies and existed a high percentage of cellular and metabolic process. The results of KEGG pathway analysis were presented in Fig. 2g and Supplementary Table S6 [37–39], most DMGs were significantly enriched in muscle-related pathways including Wnt signaling pathway, mTOR signaling pathway, and FoxO signaling pathway.

In addition, we detected dozens of well-studied muscle development related genes among DMGs, such as PITPNA, SIX2, FOXJ2, FOXK2, MYOT and so on (Supplementary Table S4). For instance, phosphatidylinositol transfer protein-α (PITPNA) is an important mediator of abnormal signaling, morphology, and function of dystrophic skeletal muscle [40]. In our m6A-seq data, the transcript of PITPNA gene carries m6A peak around 3’UTR (Supplementary Table S4). The large fraction of m6A-containing genes related to muscle development suggests a relationship between m6A modification and goose embryonic breast muscle tissues development.

### Identification of differentially expressed genes (DEGs) by RNA-seq

**Fig. 3:**
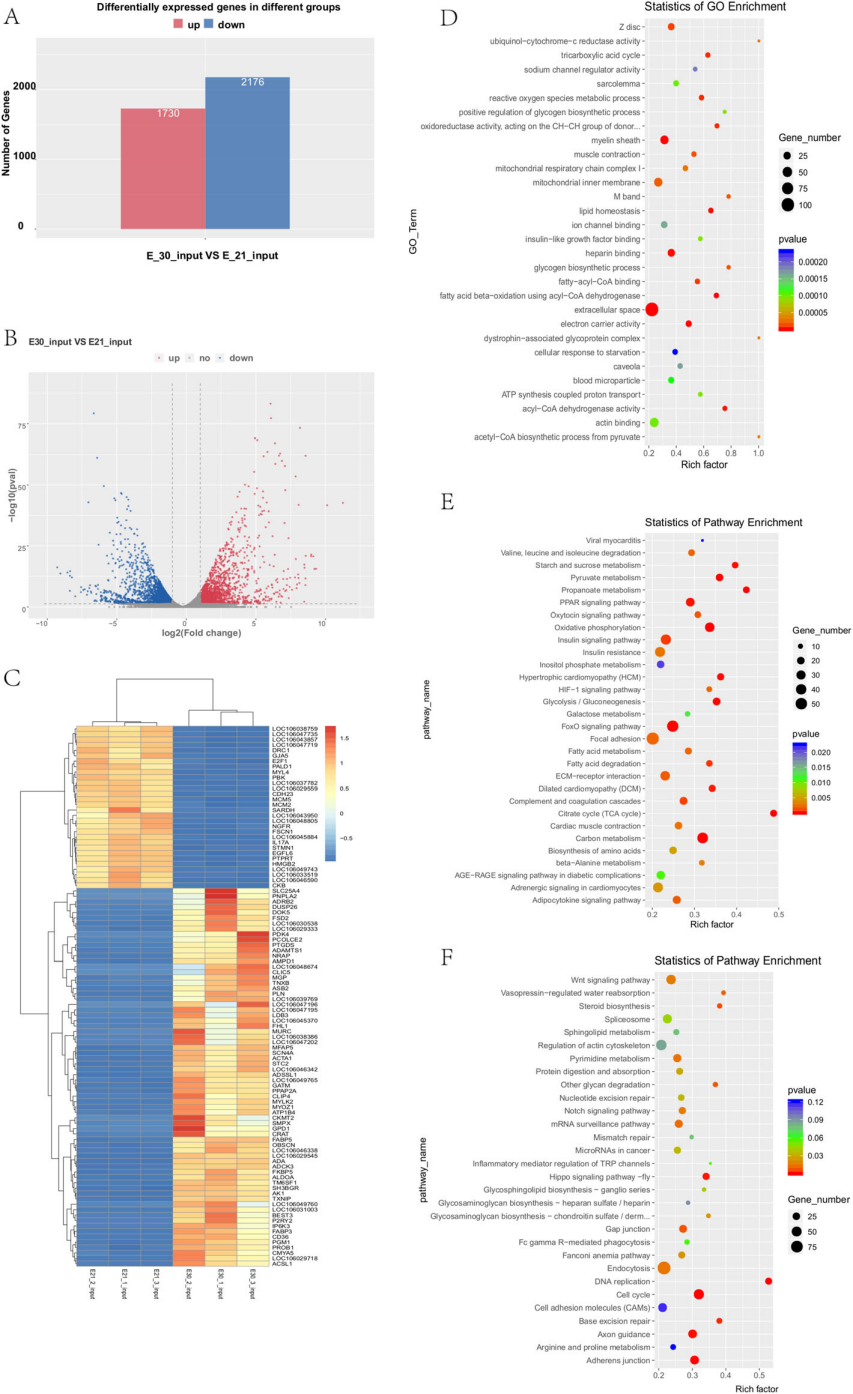
Analysis of differentially expressed genes (DEGs) between E21 and E30 of Dingan goose. **a** Number of up- and down-regulated DEGs. Red column indicates up-regulated DEGs and blue column indicate down-regulated DEGs. **b** The volcano of DEGs. **c** Heat map of DEGs. **d** Biological process of GO analysis for DEGs. **e** Pathway analysis of up-regulated DEGs. **f** Pathway analysis of down-regulated DEGs

The RNA-seq was used to describe the mRNA expression patterns between E21 and E30 embryonic breast tissues. In total of 3906 mRNAs were found significant difference between E21 and E30 including 1730 up-regulated DEGs and 2176 down-regulated DEGs (Fig. 3a; Supplementary Table S7). The volcano and the hierarchical clustering of DEGs data were shown in Fig. 3b and c.

The GO and KEGG pathway analysis were performed for DEGs. It was uncovered that DEGs between E21 and E30 were significantly enriched in biological processes including extracellular space, myelin sheath and heparin binding (Supplementary Table S8; Fig. 3d). KEGG pathway analysis showed that DEGs were mainly enriched in muscle-related pathways such as PPAR signaling pathway, FoxO signaling pathway, Fatty acid metabolism in embryonic breast tissues (Supplementary Table S9; Fig. 3e and f). From our GO functional annotation of DEGs, we found many genes, MYOG gene [32], PDK3 gene [41], IGFBP4 gene [42] have important biological roles in myoblast differentiation, ATP binding, regulation of cell growth annotations related to muscle cell development. The results above suggest DEGs may play key roles in breast muscle development of goose.

### Correlation analysis of m6A-seq and RNA-seq data

**Fig. 4:**
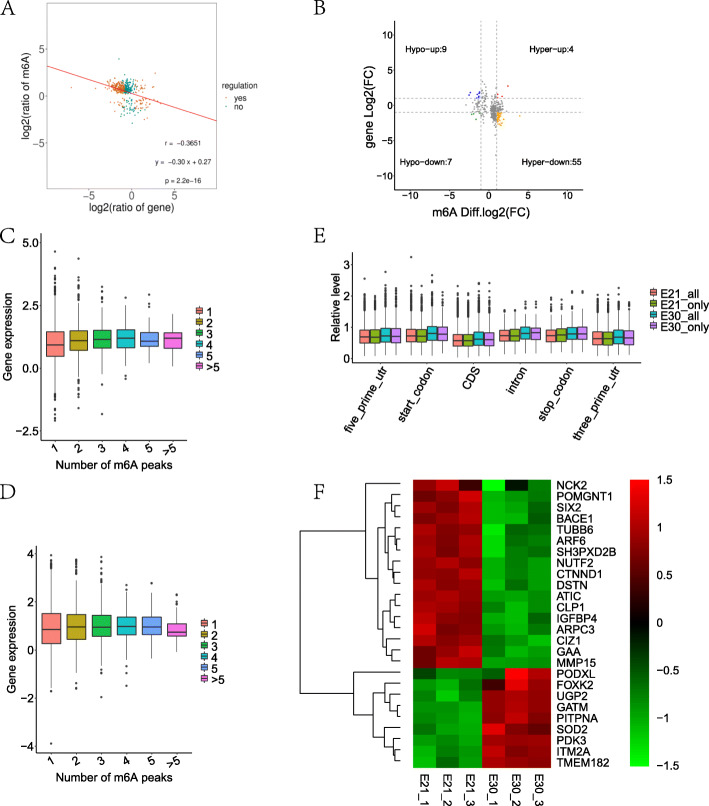
Conjoint analysis m6A-seq and RNA-seq data. **a** Negative correlation between overall m6A methylation and mRNA expression level (*r* = −0.3651; y = −0.30x + 0.27). **b** Distribution of genes with a significant change in both m6A methylation level and gene expression between E30 and E20 (DMGs). Hyper-up, Hyper-down represent genes with increased m6A methylation level and increased gene expression, genes with increased m6A methylation level and decreased gene expression, respectively. Hypo-up, Hypo-down represent genes with decreased m6A methylation level and increased gene expression, genes with decreased m6A methylation level and decreased gene expression, respectively. **c** Expression levels of genes carrying different number of m6A peaks in E21. **d** Expression levels of genes carrying different number of m6A peaks in E30. **e** The m6A modification sites and gene expression in all m6A peaks, E21-unique peaks and E30-unique peaks. **f** Heat map of 26 muscle related genes represented in Table 1

We found a negative correlation of methylated m6A level and genes expression abundance in E21 and E30 (Fig. 4a). In 328 hyper-methylated m6A sites detected by m6A-seq, we found 55 target gene with down-regulated mRNA transcripts, that is “hyper-down”. Four genes were detected to have hyper-methylated m6A sites along with up-regulated mRNA transcript, that is “hyper-up”. In parallel to 90 hypo-methylated m6A sites, we found nine targets with up-regulated mRNA transcripts, that is “hypo-up”. Seven genes were examined to have hypo-methylated m6A sites along with down-regulated mRNA transcript, that is “hypo-down” (Fig. 4b; Supplementary Table S4). In fact, we found significant differences in both m6A level and gene expression in E21 compared with E30 (Supplementary Table S4), which can be referred from the fact that the number of “hyper-down” and “hypo-up” target genes were more than those of “hyper-up” and “hypo-down” genes. Obviously, it was dominated by the negative correlation between m6A modification and mRNA abundance in E21 and E30 tissues.

We further explored the relationship of the location of m6A peaks along mRNA transcripts or the number of m6A peaks per gene with gene expression levels. As shown in Fig. 2d and e, we identified different genes owning different number of m6A peaks. Through determining the relative expression of those genes, we found that the expression levels of genes with more than one m6A sites were much higher than that of genes with one m6A sites (Fig. 4c and d). Furthermore, we divided all m6A peaks into E21-unique peaks and E30-unique peaks depending on their m6A modification sites. As a result, we found m6A genes around CDS and 3’UTR tended to have decreased expression levels (Fig. 4e).

As shown in the previous part of this paper, we obtained 397 DMGs. Further, we got 26 genes from the 397 DMGs, which were related to muscle development (Table 1). Among the 26 genes, there were eight hypo-up genes (GATM, ITM2A, PDK3, SOD2, PITPNA, UGP2, FOXK2, PODXL) and 17 hyper-down genes (NCK2, IGFBP4, NUTF2, ARPC3, CTNND1, ARF6, GAA, SIX2, TUBB6, ATIC, SH3PXD2B, POMGNT1, CIZ1, BACE1, CLP1, DSTN, MMP15). From heat map of those four groups (Fig. 4f), the expression level of these 26 picked genes was the same as in Supplementary Table S7. Considering our previous study that breast muscle growth rate of E30 was much lower than that of E21, hypo-up genes might be negative regulatory genes and hyper-down genes might be positive regulatory genes in embryonic breast muscle growth. Among these 9 hypo-up genes, glycine amidinotransferase (GATM) has been recently reported to be highly enriched in creatine-synthesis pathway in piscine muscle opposite to mammals, indicating a potential role in piscine skeletal muscle growth [43]. Similarly, a cardiotoxin-induced mouse muscle injury model was conducted to demonstrate the regulation mechanism of integral membrane protein 2A (ITM2A) in myoblast differentiation [44]. Importantly, among hyper-down genes, it has been reported that NCK2 plays a crucial role in skeletal muscle differentiation [45]. It’s worth mentioning that insulin-like growth factor binding protein 4 (IGFBP4) is also an important mediator for adipogenesis and IGF signaling in adipocytes [46].

**Table 1:** List of 26 genes that exhibit a significant change in both m6A modification and mRNA expression related to muscle development in Dingan Goose embryonic breast muscle tissues

Gene name	Pattern	Transcript ID	m6A level change					mRNA level change			Number of miRNAs
			Scaffold ID	Peak region	Peak start	peak end	Fold enrichment	Fold change	*P*-value	Strand	
GATM	Hypo-up	XM_013192762.1	146	3′ UTR	2,140,850	2,141,805	24.60	8.03	0.00	–	15
NCK2	Hyper-down	XM_013190212.1	109	3′ UTR	949,166	979,207	10.40	0.38	0.01	–	0
ITM2A	Hypo-up	XM_013192251.1	137	3′ UTR	1,084,601	1,090,313	7.64	2.54	0.00	–	13
IGFBP4	Hyper-down	XM_013201934.1	397	3′ UTR	141,061	141,330	6.26	0.27	0.00	–	12
NUTF2	Hyper-down	XM_013176707.1	24	3′ UTR	1,258,529	1,271,420	5.64	0.29	0.00	–	6
ARPC3	Hyper-down	XM_013173349.1	14	5′ UTR	2,545,265	2,548,951	5.45	0.34	0.00	–	7
PDK3	Hypo-up	XM_013183853.1	63	3′ UTR	4,436,884	4,444,805	5.16	6.03	0.00	+	53
SOD2	Hypo-up	XM_013186750.1	81	3′ UTR	3,472,567	3,479,935	4.92	2.03	0.00	+	13
CTNND1	Hyper-down	XM_013177609.1	27	3′ UTR	84,757	85,585	4.28	0.35	0.00	+	0
ARF6	Hyper-down	XM_013202366.1	448	3′ UTR	77,809	78,102	3.52	0.43	0.00	–	0
PITPNA	Hypo-up	XM_013189960.1	106	3′ UTR	265,238	265,538	3.17	2.18	0.00	+	40
GAA	Hyper-down	XM_013197895.1	238	3′ UTR	407,125	408,007	2.88	0.38	0.00	–	3
SIX2	Hyper-down	XM_013200196.1	7	3′ UTR	15,156,668	15,156,993	2.83	0.44	0.00	+	9
TUBB6	Hyper-down	XM_013179395.1	35	Exon	4,262,506	4,267,545	2.67	0.26	0.00	+	1
ATIC	Hyper-down	XM_013178608.1	32	3′ UTR	2,630,113	2,630,231	2.56	0.21	0.00	–	4
SH3PXD2B	Hyper-down	XM_013191841.1	133	Exon	1,227,288	1,227,527	2.30	0.35	0.00	+	0
UGP2	Hypo-up	XM_013196193.1	204	Exon	1,588,652	1,589,768	2.24	4.42	0.00	+	14
TMEM182	Hypo-up	XM_013190208.1	109	Exon	2,308,663	2,332,173	2.21	4.27	0.00	–	0
POMGNT1	Hyper-down	XM_013176172.1	23	3′ UTR	7,368,724	7,369,199	2.02	0.45	0.00	+	6
FOXK2	Hypo-up	XM_013198434.1	247	5′ UTR	779,207	780,467	2.00	2.77	0.00	–	0
CIZ1	Hyper-down	XM_013198642.1	251	Exon	830,940	836,624	1.97	0.32	0.00	–	0
BACE1	Hyper-down	XM_013187092.1	84	3′ UTR	2,785,912	2,786,031	1.61	0.29	0.00	+	19
CLP1	Hyper-down	XM_013177584.1	27	Exon	31,856	32,213	1.58	0.24	0.00	+	0
DSTN	Hyper-down	XM_013194951.1	180	3′ UTR	909,963	910,170	1.54	0.25	0.00	+	23
MMP15	Hyper-down	XM_013176746.1	24	Exon	1,895,816	1,896,024	1.42	0.47	0.00	–	7
PODXL	Hypo-up	XM_013185434.1	72	3′ UTR	2,083,223	2,083,492	1.35	2.06	0.00	+	1

*UTR* Untranslated region

### Correlation analysis between miRNAs-seq and m6A-seq

**Fig. 5:**
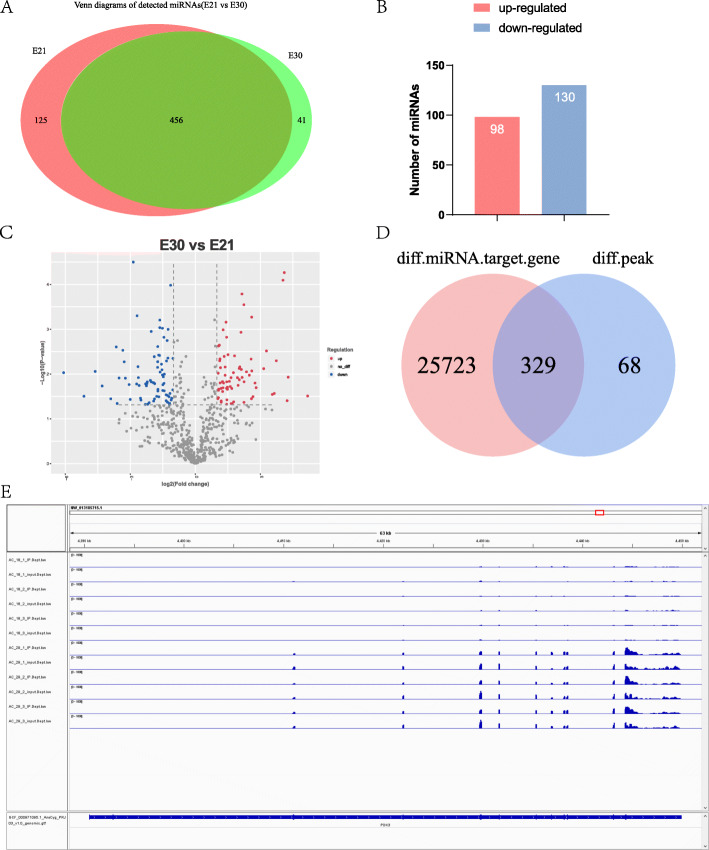
conjoint analysis m6A-seq and miRNAs-seq data. **a** Venn diagram of miRNAs expressed between E30 and E21. **b** Numbers of differentially expressed miRNAs (DEMs) between E21 and E30. **c** The volcano of differentially methylated genes (DEMs). Red, gray and blue represent up-regulated, unchanged and down-regulated miRNAs, respectively. **d** Venn diagram of the overlapped genes between target genes of DEMs and DMGs. **e** The visualization of m6A abundances in PDK3 mRNA transcripts in E30 and E21

As a member of prevail non-coding RNAs, miRNAs affect specific gene expression. In our study, we discovered 581 and 497 miRNAs in E21 and E30 (Supplementary Fig. S3A&B), respectively, and detected 456 common miRNAs (Fig. 5a). Furthermore, we found 98 up-regulated and 130 down-regulated miRNAs at *P* < 0.05 (Fig. 5b and c). Strikingly, we found 26,052 target genes of DEMs (Supplementary Table S10). To verify the potential relationship between miRNAs and m6A in embryonic muscle development of Dingan goose, we drew a venn diagram to find the shared genes between DMGs and the target genes of DEMs and found 329 genes overlapped, namely, 329 out of 397 DMGs could be potential targeted by DEMs (Fig. 5d; Table 1). Therefore, we speculated that m6A modification could be significantly manipulated by miRNAs in *Anser cygnoides orientalis* embryonic breast muscle, consisting with the proposed association in mammals [30].

In addition, we further found 10 candidate genes which belong to the 26 muscle development related genes among the 397 DMGs mentioned above and were targeted by various DEMs at the same time (called m6A-miRNA-genes in this paper) including PDK3, PITPNA, DSTN, BACE, GATM, ITM2A, SOD2, IGFBP4, GAA and TUBB6. Among the 10 m6A-miRNA-genes, PDK3, a member of pyruvate dehydrogenase kinase gene family, stood out, which was reported to specifically control functions characteristic of skeletal tissues [41]. In our data, PDK3 were targeted by 53 miRNAs (Table 1), in which miR-451 [47], miR-365 [48], miR-181a [49] were recently reported related to skeletal muscle development. The differentially methylated sites in E21 and E30 showed altered intensity around the corresponding m6A peaks, according to Integrative Genomics Viewer (IGV) software (Fig. 5e) [50]. Indeed, we should put our focus on the vital possibilities of m6A modification affecting miRNAs maturation to a large extent, or affecting the binding sites of miRNAs through reader proteins of m6A or structure modification by methylation.

## Discussion

Although m6A methylation has been widely studied in a host of species, there is no study focuses on RNA methylation in goose so far [51–53]. Namely, current studies on RNA methylation are deficient to unravel the mechanism of early embryonic growth in goose. Therefore, methylation and mechanism of m6A in goose remains largely unknown. Herein, we carried out a transcriptome-wide analysis of m6A and identified the correlation between m6A modification and muscle development related genes expression based on potential miRNAs in Dingan goose embryonic breast muscle tissues. Our study uncovered a crucial role of potential DMGs targeted by miRNAs in regulating embryonic muscle development of goose for the first time.

We chose two points of time (E21 and E30) with significantly different physiological status in embryonic breast muscles development to analyze transcriptome-wide m6A profile in Dingan goose and discovered 21,767 peaks containing 12,073 genes. Subsequent analysis showed the methylated m6A peaks tend to enrich on the CDS and start codon. This profile was not consistent with mammals that the m6A around stop codon is significantly enriched [29], suggesting that the methylation profiles of m6A are unique in goose transcriptome. The importance of m6A methylation has been verified in various biological processes of self-renewal, differentiation, early embryonic development and gene expression [54]. In chicken follicles, transcriptome-wide m6A had been carried out and suggested a negative correlation between m6A methylation enrichment and gene expression levels [28]. However, the negative correlation between m6A modification and gene expression in our data is contrary to the case of *Arabidopsis thaliana* where m6A deposition and mRNA abundance were positively correlated [23]. We further found the expression levels of “hypo-up” and “hyper-down” genes were higher than those of “hypo-down” and “hyper-up” genes in Dingan goose embryonic muscle tissues, which consisted with the results of chicken and implied a possible regulatory mechanism of m6A methylation in goose breast muscle development. In addition, we found 397 DMGs mainly enriched in terms of positive regulation of GTPase activity, protein phosphorylation in GO-enrichment analysis, meanwhile enriched in Wnt signaling pathway, mTOR signaling pathway, and FoxO signaling pathway in KEGG pathway analysis.

We discovered 3906 DEGs were significantly enriched in extracellular space, myelin sheath and heparin binding from our RNA-seq data. KEGG pathway analysis showed that DEGs in significantly enriched pathways involved in muscle-related pathways such as PPAR signaling pathway, FoxO signaling pathway, Fatty acid metabolism. From our GO and KEGG analysis, both DMGs and DEGs involved in not only skeletal development (such as Wnt signaling pathway and mTOR signaling pathway) but also fat deposition (such as PPAR signaling pathway, FoxO signaling pathway, Fatty acid metabolism). Further, we also found MYOG gene, PDK3 gene, IGFBP4 gene have distinct biological processes related to muscle cell development, suggesting a potential mechanism that functional genes regulating goose embryonic skeletal muscle development.

Mature miRNAs regulated specific target mRNAs expression based on methyltransferase-like 3 (METTL3) and RNA binding protein HNRNPA2B1 in m6A methylation process [55, 56]. Herein, we identified 228 DEMs and correlated all of the DEMs’ target genes with DMGs to picked out muscle-related m6A-miRNA-genes. As a result, we selected 10 m6A-miRNA-genes consisting of 5 hypo-up genes (PDK3, PITPNA, GATM, ITM2A and SOD2) and 5 hyper-down genes (DSTN, BACE1, IGFBP4, GAA and TUBB6). Because the m6A-miRNA-genes were the overlapped genes by DMGs and target genes of DEMs and breast muscle development in E21 than that in E30, the 5 hypo-up genes were probably the potential negative regulators and 5 hyper-down genes were probably the potential positive regulators of breast muscle development in Dingan goose. After literatures searching, we did not find their related m6A functions for the 10 genes. However, they are all involved in regulation of skeletal muscle development. PITPNA are assumed being as a protective genetic modifier in curing muscular dystrophy dogs [57] and PITPNA knockdown by lentiviral shRNA increased both pAkt expression and myoblast fusion index [40]. In addition, BACE was found primarily in pancreas, liver, and muscle and its expression pattern is consistent with the tendency of myogenic cells to differentiate into myofibroblasts [58]. Especially, PDK3, performed an important role in muscle development [38], and our visualization results showed that there were differentially methylated peaks on the PDK3 mRNA transcript in E21 and E30, confirming the validity of our analysis of m6A and miRNA data.

## Conclusion

This study, for the first time, uncovered transcriptome-wide m6A modification pattern affecting embryonic breast muscle development in Dingan goose. Our m6A map revealed the characteristics of m6A modification distribution in goose transcriptome. We also categorized the DMGs and discovered a negative correlation between m6A methylation and gene expression. We also picked out 10 potential m6A-miRNA-genes which were tightly associated with breast muscle development through affecting the m6A modification levels of their target gene. This comprehensive analysis provides a potential clue between m6A modification and muscle differentiation genes expression based on regulatory miRNAs.

## Materials and methods

### Ethics statement

All geese were obtained from the Institute of Animal Science & Veterinary Medicine, Hainan Academy of Agricultural Sciences (IASVM-HAAS, Haikou, China). Ethical approval (reference number: IASVMHAAS-AE-202022) was conferred by the animal ethics committee of IASVM-HAAS, which is responsible for animal welfare. All experimental protocols were conducted in accordance with guidelines established by the Ministry of Science and Technology (Beijing, China).

### Anatomy experiment

The embryos of Dingan goose were provided by the breeding farm of Dingan goose of Nanhua Dingan goose Breeding Ltd. A total of 200 selected eggs were incubated at 37.2 ~ 38.5 °C with a humidity of ~ 87%. From E15 to E30, 10 eggs were taken randomly every day. The embryos were extracted and weighed, and the breast muscles were stripped and weighed. The rates of breast muscles were calculated using the formulas as follows: PMR = PMWn/BWn and LMR = LMWn/BWn, where PMR and LMR represent the chest and limb muscle rates, PMWn and LMWn indicate breast muscle weights on day n after hatching, and BWn means body weight on day n after hatching.

### Collection of breast muscle samples

In order to analysis the transcriptome-wide m6A level in Dingan goose, six embryonic breast muscle tissues of E21 and E30 day during hatching were selected as two different treatment groups with three biological randomly replicates for each group. Each healthy embryo was taken out from the eggs, and then the breast muscle was peeled off. The samples of breast muscles were divided into three pieces after breast muscles were stripped. The first and second breast muscle pieces were snap-frozen in liquid nitrogen and kept at − 80 °C till to further m6A-seq and qRT-PCR analysis, respectively. The third piece of breast muscle, which was emerged in 4% paraformaldehyde immediately, was used for histological section (hematoxylin and eosin (HE) staining).

### Morphologic analysis of breast muscles

The chest and limb muscle samples at six embryonic stages (E15, E18, E21, E24 E27, and E30) were rinsed with running water, followed by dehydration in a gradient of ethanol dilutions. Dehydrated samples were treated with xylene for three times and embedded with paraffin, followed by preparation into 4 μm sections. A paraffin ribbon was placed at 40 °C in a water bath. Sections were mounted onto slides and then air-dried for 30 min, followed by incubation at 45 °C overnight. Dewaxation was carried out with xylene for two times, followed by hydration in a series of ethanol solutions, and finally the sections were washed in distilled water for 5 min. Slices were stained with hematoxylin & eosin (HE). A digital microscope (Nikon) was employed to acquire pictures. Five individuals were examined for each group, and five sections were assessed for each individual with five randomly selected fields per section. Averagely, 125 myofibers were assessed for each bird. The myofiber diameter (MFD) and mean number of myofibers in the unit area (MFN) were determined by Image-Pro Plus 6.0 software (Media Cybernetics, Bethesda, MD).

### Expression profiles of MyoD, MyoG and MSTN

The breast muscle samples were immediately stripped, snap-frozen in liquid nitrogen and kept at − 80 °C prior to further analysis after obtaining. Total RNA was extracted using Trizol (Invitrogen, USA) according to the manufacturer’s instructions. The quantitative real-time reverse-transcription polymerase chain reaction (qRT-PCR) was performed using the SYBR PrimeScript RT-PCR Kit (TaKaRa, Japan) on an iCycler IQ5 Multicolor Real-Time PCR Detection System (Bio-Rad, USA). The expressions of MyoD, MyoG and MSTN were determined, and β-actin was employed as housekeeping gene. qRT-PCR was conducted in a 25 μL reaction system consisting of 1 μL of cDNA template, 12.5 μL of SYBR Premix Ex-Taq, 10.5 μL of sterile water, and 0.5 L of gene-specific primer (Supplementary Table S11). Briefly, after an initial denatuartion step at 95 °C for 30 s, amplifications were carried out with 40 cycles at a melting temperature of 95 °C for 10 s and an annealing temperature at 60 °C for 40 s. In order to confirm product specificity, a melting curve analysis was performed for each run. Each experiment was conducted in triplicate. Moreover, plasmids containing corresponding cDNA were serially diluted from 10 to 1 to 10–8 to generate gene-specific standard curves. Above-mentioned plasmid dilutions were employed as the PCR templates to determine the amplification efficiency of each primer set. Standard curve data (R2, slope and efficiency) were given in Supplementary Table S11.

### RNA extraction and fragmentation

In our study, total RNA of E21 and E30 breast muscle tissues were isolated and purified by Trizol reagent (Invitrogen, Carlsbad, CA, USA) following the manufacturer’s procedure. The RNA amount and purity of each sample was quantified using NanoDrop ND-1000 (NanoDrop, Wilmington, DE, USA). The RNA integrity was assessed by Bioanalyzer 2100 (Agilent, CA, USA) with RIN number > 7.0, and confirmed by electrophoresis with denaturing agarose gel. Poly (A) RNA is purified from 50 μg total RNA using Dynabeads Oligo (dT)25–61,005 (Thermo Fisher, CA, USA) by two rounds of purification. Then the poly(A) RNA was fragmented into small pieces using Magnesium RNA Fragmentation Module (NEB, cat. e6150, USA) under 86 °C for 7 min, and subjected to immunoprecipitation and sequencing.

### m6A immunoprecipitation, library construction and sequencing

mRNA was randomly fragmented into 100-nucleotide-long fragments, and then the cleaved RNA fragments were incubated for 2 h at 4 °C with m6A-specific antibody (No. 202003, Synaptic Systems, Germany) in IP buffer (50 mM Tris-HCl, 750 mM NaCl and 0.5% Igepal CA-630). The mixture was then incubated protein-A breads at 4 °C for 2 h. After washing with, bound RNA was eluted from the protein-A breads with 0.5 mg/mL N6-methyladenosine in IP buffer (1 × IP buffer and 6.7 mM m6A) and precipitated by ethanol. The RNA was used to conduct m6A-seq library with Tru standard mRNA Sample Prep Kit (Illumina) according to a published protocol [59]. IP RNA and input RNA were sequenced on Illumina Novaseq™ 6000 platform (LC Bio Technology CO., Ltd. Hangzhou, China) with 2 × 150 bp paired-end reads in PE150.

### Bioinformatics analysis of m6A-seq and RNA-seq data

Cutadapt (v 1.10) [60] and Perl scripts in house were used to remove the contained adaptor contamination, low quality bases and undetermined bases. Then, we used the fastp software (https://github.com/OpenGene/fastp) to verify the sequence quality of IP and input of E21 and E30. We used HISAT2 (v 1.0; http://daehwankimlab.github.io/hisat2) [61] to map valid reads to the reference genome of *Anser cyhnoides orientalis* (v 1.0; https://www.ncbi.nlm.nih.gov/genome/?term=Anser+cygnoides) published on NCBI website.

Peak calling on genome-wide was provided with R package exomePeak (https://bioconductor.org/packages/exomePeak) [34] using mapped reads of IP and input libraries. Peaks were examined by Poisson distribution matrix with default parameter (*P* < 0.05). Both of the common peaks and unique peaks were annotated by ChIPseeker software (v 1.0; https://bioconductor.org/packages/ChIPseeker) [62]. MEME (v 1.0; http://meme-suite.org) [63] and HOMER (v 4.1; http://homer.ucsd.edu/homer/motif) [64] were used for de novo and known motif finding followed by localization of the motif with respect to peak summit. Then StringTie (v 1.0; https://ccb.jhu.edu/software/stringtie) [65] was accessed to quantify the expression level of all genes and transcripts containing in called peaks from input library by calculating FPKM (total exon fragments /mapped reads (millions) × exon length (kB)). Similarly, The DEGs were selected with log2 (fold change) > 2 or log2 (fold change) < 0.5 and *p* value < 0.05 by R package edgeR (https://bioconductor.org/packages/edgeR) [66] and marked with significant parameter. Gene enrichment analysis was performed by GO Ontology (GO) (http://www.geneontology.org/) and Kyoto Encyclopedia of Genes and Genomes (KEGG) (http://www.kegg.jp/).

### Correlation analysis of m6A-seq and RNA-seq data

To comprehensively compare the relationship of methylated m6A level and genes expression abundance, the relationship of methylated m6A level and genes expression abundance, relationship of the location of m6A peaks along mRNA transcripts and the number of m6A peaks per gene with gene expression levels, we performed correlation analysis of m6A-seq and RNA-seq data. Through the analysis of m6A-seq and RNA-seq data, we obtained the differentially methylated m6A peaks in abundance and DEGs. We divided the differentially methylated m6A peaks into up abundant methylated m6A sites (higher methylated m6A sites in E30 than that in E21, hyper-methylated m6A sites) and down abundant methylated m6A sites (higher methylated m6A sites in E21 than that in E30, hypo-methylated m6A sites). Similarly, DEGs were also divided into up-regulated genes (higher expression levels in E30 than that in E21) and down-regulated genes (higher expression levels in E21 than that in E30). We overlapped hyper- and hypo-methylated m6A sites with up- and down-regulated genes, and then obtained the up-regulated genes with hyper-methylated m6A sites (hyper-up), down-regulated genes with hyper-methylated m6A sites (hyper-down), up-regulated genes with hypo-methylated m6A sites (hypo-up), down-regulated genes with hypo-methylated m6A sites (hypo-down).

### Library preparation, sequencing and data analysis of miRNAs-seq

About 1 μg of the total RNA was used to conduct the small RNA library with TruSeq Small RNA Sample Prep Kits (Illumina, San Diego, USA), according to the manufacturer’s protocol. While the small RNA library was performed on Illumina Hiseq2500 (LC Bio Technology CO., Ltd. Hangzhou, China) and generated 50 bp single-end reads. RNA 6000 Nano LabChip Kit (Agilent, CA, USA). ACGT101-miR (LC Sciences, Houston, Texas, USA) was used to remove the adapter, junk and low-quality sequence of small RNA. Then, the rest of sequence was aligned to Rfam database (http://rfam.xfam.org), and Repbase Database (http://www.girinst.org/education/index.html) to filtering the non-miRNAs and repeat associate sRNA sequence, respectively. We mapped the 18 ~ 26-nucleotide - long sequence to *Anser cygnoides orientalis* in miRBase (ftp://mirbase.org/pub/mirbase/CURRENT/) by a BLAST search to categorize the generated miRNAs to known miRNAs (mapping to specific species’ mature miRNAs in hairpin arms) and novel miRNAs (not mapping to specific species’ mature miRNAs in hairpin arms). RNAfold software (University of Vienna, Vienna, Austria) (http://rna.tbi.univie.ac.at/cgi-bin/RNAWebSuite/RNAfold.cgi) was used to predicted the unmapped sequences and the hairpin RNA structure containing sequences. In contrary with novel miRNAs, we predicted its pri-miRNAs sequence by extending to 80 nt bases on corresponded comparison sites of genome.

DEMs were selected by T-test method (http://en.wikipedia.org/wiki/Student‘s_t-test) to compare the E21 and E30 (*P* < 0.05). Heatmap is used to analysis the cluster pattern in different control set with log10 value. Target genes of DEMs with significant difference were predicted by TargetScan algorithm [67–69] with default parameter and miRanda algorithm [70, 71] (Max_Energy<− 10) according to the score standard. Finally, the overlaps predicted by both algorithms were calculated.

### Integrative analysis among m6A-seq, mRNA-seq and miRNA-seq data

After we obtained the differentially methylated m6A peaks in abundance and DEGs, we searched the DEGs to check whether a DEG contains one or more differentially methylated m6A sites. If a DEG one or more differentially methylated m6A sites, it was called the differentially methylated gene (DMGs). Through analysis of miRNAs, we got the differentially miRNAs (DEMs). The targets of DEMs were then predicted. Finally, we overlapped the DMGs and the targets of DEMs and obtained the DMGs targeted by various DEMs (called m6A-miRNA-genes in this paper). The m6A-miRNA-genes can be used for the further analysis in the future.

## Supplementary Information

**Additional file 1: Supplementary Figure S1.** QPCR assay result of muscle development related genes from E15 to E30 in Dingan Goose embryonic breast muscle. (**A)** MSTN gene expression from E15 to E30. **(B)** MYOG gene expression from E15 to E30. **(C)** MYOD gene expression from E15 to E30. **Supplementary Figure S2.** Valid reads from E21 and E30 were mapped to exon, intron and intergenic. **Supplementary Figure S3.** Overview of miRNAs expression from miRNA-seq in both E21 and E30. **(A) V**enn diagrams of detected miRNAs in E21. Red, green, blue represent three biological repeats. **(B)** Venn diagrams of detected miRNAs in E30. Red, green, blue represent three biological repeats.

**Additional file 2: Supplementary Table S1.** Summary of sequence data and read alignment statistics. **Supplementary Table S2.** Common peaks and unique peaks between IP and input. **Supplementary Table S3.** The motif sequence for m6A-containing peak regions.

**Additional file 3: Supplementary Table S4.** Differentially methylated genes (DMGs) in E21 vs. E30 groups.

**Additional file 4: Supplementary Table S5.** Differetially methylated genes in GO analysis.

**Additional file 5: Supplementary Table S6.** Differetially methylated genes in KEGG pathway analysis.

**Additional file 6: Supplementary Table S7.** Differetially expressed genes in RNA-seq.

**Additional file 7: Supplementary Table S8.** Differetially expressed genes in GO analysis.

**Additional file 8: Supplementary Table S9.** Differetially expressed genes in KEGG pathway analysis.

**Additional file 9: Supplementary Table S10.** Differetially expressed miRNAs in E30 and E21 groups.

**Additional file 10: Table S11.** Primer sequences and standard curve data for real-time quantitative PCR analysis.

## Data Availability

Sequences are available from GenBank with the Bioproject accession numbers from SRR13052489 to SRR13052506.
