# Supplementary material for: Transcriptome-wide study revealed m6A regulation of embryonic muscle development in Dingan goose (Anser cygnoides orientalis)
Source: BMC Genomics. 2021 Apr 14;22:270. doi: 10.1186/s12864-021-07556-8 (PMC8048326; doi:10.1186/s12864-021-07556-8)
Supplement: Supplementary file 1 — Additional file 1: Supplementary Figure S1. QPCR assay result of muscle development related genes from E15 to E30 in Dingan Goose embryonic breast muscle. (A) MSTN gene expression from E15 to E30. (B) MYOG gene expression from E15 to E30. (C) MYOD gene expression from E15 to E30. Supplementary Figure S2. Valid reads from E21 and E30 were mapped to exon, intron and intergenic. Supplementary Figure S3. Overview of miRNAs expression from miRNA-seq in both E21 and E30. (A) Venn diagrams of detected miRNAs in E21. Red, green, blue represent three biological repeats. (B) Venn diagrams of detected miRNAs in E30. Red, green, blue represent three biological repeats. [file 12864_2021_7556_MOESM1_ESM.zip › Legend of Fig.S1-3.docx]

**Supplementary Figure S1. QPCR assay result of muscle development related genes from E15 to E30 in Dingan Goose embryonic breast muscle.** **(A)** MSTN gene expression from E15 to E30. **(B)** MYOG gene expression from E15 to E30. **(C)** MYOD gene expression from E15 to E30.

**Supplementary Figure S2. Valid reads from E21 and E30 were mapped to exon, intron and intergenic.**

**Supplementary Figure S3. Overview of miRNAs expression from miRNA-seq in both E21 and E30. (A)** **V**enn diagrams of detected miRNAs in E21. Red, green, blue represent three biological repeats. **(B)** Venn diagrams of detected miRNAs in E30. Red, green, blue represent three biological repeats.
