# Supplementary material for: Transcriptome-wide study revealed m6A regulation of embryonic muscle development in Dingan goose (Anser cygnoides orientalis)
Source: BMC Genomics. 2021 Apr 14;22:270. doi: 10.1186/s12864-021-07556-8 (PMC8048326; doi:10.1186/s12864-021-07556-8)
Supplement: Supplementary file 2 — Additional file 2: Supplementary Table S1. Summary of sequence data and read alignment statistics. Supplementary Table S2. Common peaks and unique peaks between IP and input. Supplementary Table S3. The motif sequence for m6A-containing peak regions. [file 12864_2021_7556_MOESM2_ESM.docx]

**Supplementary Table S1.** Summary of sequence data and read alignment statistics

|  | Sample ID | Raw Reads | Valid Reads | Mapped reads | m6A peaks/m6A modified genes | Expressed gene |
| --- | --- | --- | --- | --- | --- | --- |
| IP-seq | E21_1 | 64949554 | 56297378 | 40514787(71.97%) | 12770/6650 |  |
|  | E21_2 | 68125042 | 62167202 | 44550618(71.66%) |  |  |
|  | E21_3 | 72385584 | 65007012 | 46155327(71.00%) |  |  |
|  | E30_1 | 69531456 | 61693400 | 46028367(74.61%) | 8997/5423 |  |
|  | E30_2 | 73434100 | 65341902 | 46244533(70.77%) |  |  |
|  | E30_3 | 83793834 | 78899918 | 58262058(73.84%) |  |  |
| Input-seq | E21_1 | 93014318 | 80820066 | 48043532(59.45%) |  | 12741 |
|  | E21_2 | 88316634 | 78117834 | 45850241(58.69%) |  | 11893 |
|  | E21_3 | 92642996 | 82655274 | 49110299(59.42%) |  | 12857 |
|  | E30_1 | 92287266 | 85926726 | 53073013(61.77%) |  | 10737 |
|  | E30_2 | 82753564 | 68958750 | 43904293(63.67%) |  | 12120 |
|  | E30_3 | 79374280 | 67309076 | 41713525(61.97%) |  | 12143 |

**Note:** E21_1, E21_2 and E21_3 mean the sample 1, sample 2 and sample 3 of embryonic breast muscle of 21^th^ day from *Anser cygnoides orientalis.* E30_1, E30_2 and E30_3 mean the sample 1, sample 2 and sample 3 of embryonic breast muscle of 30^th^ day from *Anser cygnoides orientalis.*

**Supplementary Table S2.** Common peaks and unique peaks between IP and input.

| Tissue | m6A peaks | Common peaks | Unique peaks | | | | | | |
| --- | --- | --- | --- | --- | --- | --- | --- | --- | --- |
|  |  |  | Total | Stop codon | Start codon | 3’ UTR | 5’ UTR | CDS | Intron |
| E21 | 8308 | 4462 | 12770 | 98 | 1043 | 819 | 1905 | 5391 | 120 |
| E30 | 4535 |  | 8997 | 66 | 685 | 540 | 1076 | 3165 | 57 |

**Note:** E21 means the sample of embryonic breast muscle of 21^th^ day from *Anser cygnoides orientalis.* E30 means the sample of embryonic breast muscle of 30^th^ day from *Anser cygnoides orientalis.*

**Supplementary Table S3.** The motif sequence for m6A-containing peak regions.

|  | Motif | *P*-value | % of Target | % of Background |
| --- | --- | --- | --- | --- |
| E21_IP vs. E21_input | 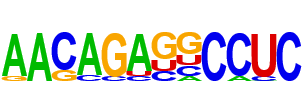 | 1e-14 | 1.93% | 0.08% |
|  | 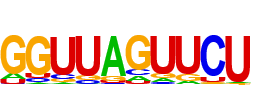 | 1e-12 | 6.20% | 1.67% |
|  | 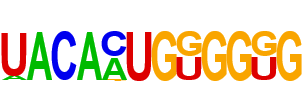 | 1e-12 | 1.10% | 0.01% |
| E30_IP vs. E30_input | 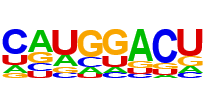 | 1e-16 | 22.62% | 11.14% |
|  | 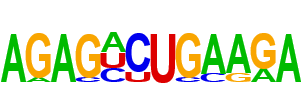 | 1e-13 | 2.23% | 0.13% |
|  | 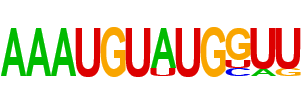 | 1e-13 | 1.04% | 0.01% |

**Note:** Analysis was performed by using Hypergeometric Optimization of Motif Enrichment software. E21, E30, mean the sample of embryonic breast muscle of 21^th^ and 30^th^ day from *Anser cygnoides orientalis,* respectively.
