# Supplementary material for: Transcriptome-wide study revealed m6A regulation of embryonic muscle development in Dingan goose (Anser cygnoides orientalis)
Source: BMC Genomics. 2021 Apr 14;22:270. doi: 10.1186/s12864-021-07556-8 (PMC8048326; doi:10.1186/s12864-021-07556-8)
Supplement: Supplementary file 10 — Additional file 10: Table S11. Primer sequences and standard curve data for real-time quantitative PCR analysis. [file 12864_2021_7556_MOESM10_ESM.docx]

**Table S11: Primer sequences and standard curve data for real-time quantitative PCR analysis.**

| Target gene | Primer sequence (5'-3') | Product size (bp) | R^2a^ | Slope^b^ | Efficiency^c^ |
| --- | --- | --- | --- | --- | --- |
| β-Actin | Forward: GCTATGTCGCCCTGGATTTC | 168 | 0.999 | -3.356 | 0.983 |
|  | Reverse: ACAGGACTCCATACCCAAGAA |  |  |  |  |
| MyoD | Forward: GGCTCAGCAAGGTCAACG | 94 | 0.996 | -3.336 | 0.970 |
|  | Reverse: CTCTCGATGTAGCGGATGG |  |  |  |  |
| MyoG | Forward: CGGATCACCTCCTGCCTGA | 87 | 0.997 | -3.353 | 0.951 |
|  | Reverse: CGTCCTCTAACGGCGATGCT |  |  |  |  |
| MSTN | Forward: CTGGTATTTGGCAGAGTATTG | 80 | 0.997 | -3.436 | 0.979 |
|  | Reverse: CTCGTCCATTCTCATCAAAAG |  |  |  |  |

NOTE:

^a^ Regression coefficient of standard curve.

^b^The slope of standard curve.

^c^ Amplification efficiency of PCR.
